# Clinical characteristics and outcomes of patients hospitalized with heart failure with preserved ejection fraction and low NT-proBNP levels

**DOI:** 10.1097/MD.0000000000036351

**Published:** 2023-11-24

**Authors:** Yu-Yi Chen, Lin Liang, Peng-Chao Tian, Jia-Yu Feng, Li-Yan Huang, Bo-Ping Huang, Xue-Mei Zhao, Yi-Hang Wu, Jing Wang, Jing-Yuan Guan, Xin-Qing Li, Jian Zhang, Yu-Hui Zhang

**Affiliations:** a Heart Failure Center, State Key Laboratory of Cardiovascular Disease, Fuwai Hospital, National Center for Cardiovascular Diseases, Chinese Academy of Medical Sciences & Peking Union Medical College, Beijing, China.

**Keywords:** heart failure with preserved ejection fraction, independent predictors, N-terminal pro-B-type natriuretic peptide, prognosis

## Abstract

The aim of this study was to investigate the clinical characteristics and prognosis of patients hospitalized with heart failure with preserved ejection fraction (HFpEF) and low N-terminal pro-B-type natriuretic peptide (NT-proBNP) levels. Seven hundred ninety consecutive patients hospitalized with HFpEF from 2006 to 2017 were enrolled. Clinical characteristics and outcomes were compared between low NT-proBNP group (<300 ng/L) and elevated NT-proBNP group (≥300 ng/L). 108 HFpEF patients (13.7%) presented with low NT-proBNP levels. Age, body mass index, atrial fibrillation, New York Heart Association functional class, and albumin were independent predictors of low NT-proBNP levels in HFpEF patients. During the median follow-up duration of 1103 days, 11 patients (10.2%) in low NT-proBNP group suffered from primary endpoint event. Elevated NT-proBNP group had a higher risk of all-cause death or heart transplantation than low NT-proBNP group (adjusted HR [95%CI]: 2.36 [1.24,4.49], *P* = .009). Stratified analyses showed that the association between NT-proBNP (elevated NT-proBNP group vs low NT-proBNP group) and risk of all-cause death or heart transplantation was stronger in non-atrial fibrillation patients than in atrial fibrillation patients (*P* value for interaction = .025). Furthermore, the associations between NT-proBNP and risk of all-cause death or heart transplantation were stronger in younger and male patients than in older and female patients. However, both subgroups only reached borderline significant (*P* values for interaction = .062 and .084, respectively). Our findings suggest that low NT-proBNP levels were common in patients hospitalized with HFpEF. Patients with HFpEF and low NT-proBNP levels had a better prognosis than those with elevated NT-proBNP levels, particularly in younger, male, and non-atrial fibrillation patients.

## 1. Introduction

Heart failure with preserved ejection fraction (HFpEF) is a rapidly growing public health issue, associated with worse quality of life and increased mortality. Approximately 64.3 million patients present with heart failure worldwide, and patients with HFpEF account for up to 50%.^[1,2]^ One meta-analysis reported that 1-year, 2-year, 5-year, and 10-year survival rates of heart failure were 86.5%, 72.6%, 56.7%, and 34.9%, respectively. And the prognosis of patients with HFpEF was as poor as those with heart failure with reduced ejection fraction.^[3]^ However, effective treatments on patients with HFpEF are still uncertain.^[4,5]^

Natriuretic peptide has been widely used for prediction, diagnosis, and prognosis evaluation of heart failure.^[4,5]^ However, multiple stimuli including mechanical stretch, ischemic injury, endothelin-1, interleukin-1β, β-adrenergic agonist, thyroid hormone and so on are associated with the increase of natriuretic peptide levels,^[6]^ and natriuretic peptide levels are easily affected by cardiac and noncardiac confounders such as body mass index (BMI), renal function, atrial fibrillation, New York Heart Association (NYHA) functional class, left ventricular ejection fraction (LVEF), and so on.^[7,8]^ A substantial proportion of symptomatic patients with HFpEF present with low natriuretic peptide levels in real world. One study reported that patients with low N-terminal pro-B-type natriuretic peptide (NT-proBNP) levels accounted for 13% in all patients hospitalized with HFpEF. Patients with HFpEF and low NT-proBNP levels were younger and had higher BMI and less atrial fibrillation and structural heart disease than those with elevated NT-proBNP levels. And 30-day readmission and 1-year survival rates were comparable between the 2 groups.^[9]^ However, I-PRESERVE study reported that patients with HFpEF and elevated NT-proBNP levels had a higher risk of cardiovascular events compared to those with low NT-proBNP levels.^[10]^

Up to date, studies on the association between low natriuretic peptide levels and HFpEF are scarce. This retrospective cohort study aimed to investigate the incidence of patients hospitalized with HFpEF and low NT-proBNP levels, independent predictors of low NT-proBNP levels in patients with HFpEF, and prognosis of patients with HFpEF and low NT-proBNP levels compared to those with elevated NT-proBNP levels.

## 2. Methods

### 2.1. Study population

Consecutive patients hospitalized with HFpEF in the heart failure center of Fuwai Hospital (Beijing, China) from December 2006 to December 2017 were enrolled. The diagnosis of HFpEF included typical symptoms (e.g., dyspnea, fatigue, and ankle edema) and/or specific signs (e.g., elevated jugular venous pressure, displaced apical impulse, and pulmonary crackles) of heart failure, LVEF ≥ 50%, objective evidences of cardiac structural and/or functional abnormalities (e.g., ventricular hypertrophy, cardiac chamber enlargement, or valvular stenosis or regurgitation), objective evidences of left ventricular diastolic dysfunction or increased left ventricular filling pressure [e.g., E/e′ ratio ≥ 13, E/A ratio < 1, estimated pulmonary artery systolic pressure > 35 mm Hg, elevated NT-proBNP level, or pulmonary congestion], and exclusion of other non-heart failure factors, which 2 cardiac specialists confirmed according to Chinese heart failure management guideline.^[11]^ Exclusion criteria included: (1) patients with non-heart failure, heart failure with reduced ejection fraction, or heart failure with mildly reduced ejection fraction; (2) lacking baseline NT-proBNP concentrations; (3) lost to follow-up. Patients with HFpEF were classified into low NT-proBNP group (<300 ng/L) and elevated NT-proBNP group (≥300 ng/L) according to baseline NT-proBNP levels. Baseline clinical, laboratory and echocardiographic data were collected as well as prescribed medication. This clinical study was approved by the Ethics Committee of Fuwai Hospital. All enrolled patients have signed the written informed consent.

### 2.2. Follow-up and primary endpoint event

After patients were discharged from hospital, clinical visits or telephone interviews were carried out for each patient at 3-month, 6-month, 1-year, and every 6-month interval thereafter. The primary endpoint event was the composite of all-cause death or heart transplantation. Follow-up time was calculated from admission time to the time of primary endpoint event or the last follow-up time.

### 2.3. NT-proBNP measurements

All samples were collected within 24 hours after patients admitted into heart failure center. Plasma NT-proBNP levels were measured with an electrochemiluminescence immunoassay (Roche Diagnostics, Germany) at the clinical laboratory of Fuwai Hospital. The testing range of NT-proBNP assay was between 5 and 30,000 ng/L, and the intra-assay coefficient of variation was <5%.

### 2.4. Statistical analysis

Data were presented with mean and standard deviation or median and interquartile range for continuous variables and n and percentage for categorical variables. Comparisons for normal distributed data were performed with independent sample *T* test. Mann–Whitney *U* test was used for comparisons of non-normal distributed data. Categorical data were compared with Chi-Square test. Multivariate logistic regression analysis was performed to detect independent predictors of low NT-proBNP levels in patients with HFpEF. For time-to-event data, the relationship of elevated NT-proBNP group and low NT-proBNP group with primary endpoint event was performed with Kaplan–Meier analysis and log-rank test. Adjusted analysis for the primary endpoint event was performed with multivariate Cox regression analysis. Similarly, adjusted analyses for the primary endpoint event in subgroups including age, sex, BMI, hypertension, diabetes mellitus, coronary artery disease, atrial fibrillation, estimated glomerular filtration rate (eGFR), LVEF, and NYHA functional class were performed with multivariate Cox regression analyses, and their interactions were tested. *P*-values of <.05 were considered statistically significant. All analyses were performed with SPSS version 25.0 and R version 4.2.1.

## 3. Results

**Figure 1.**
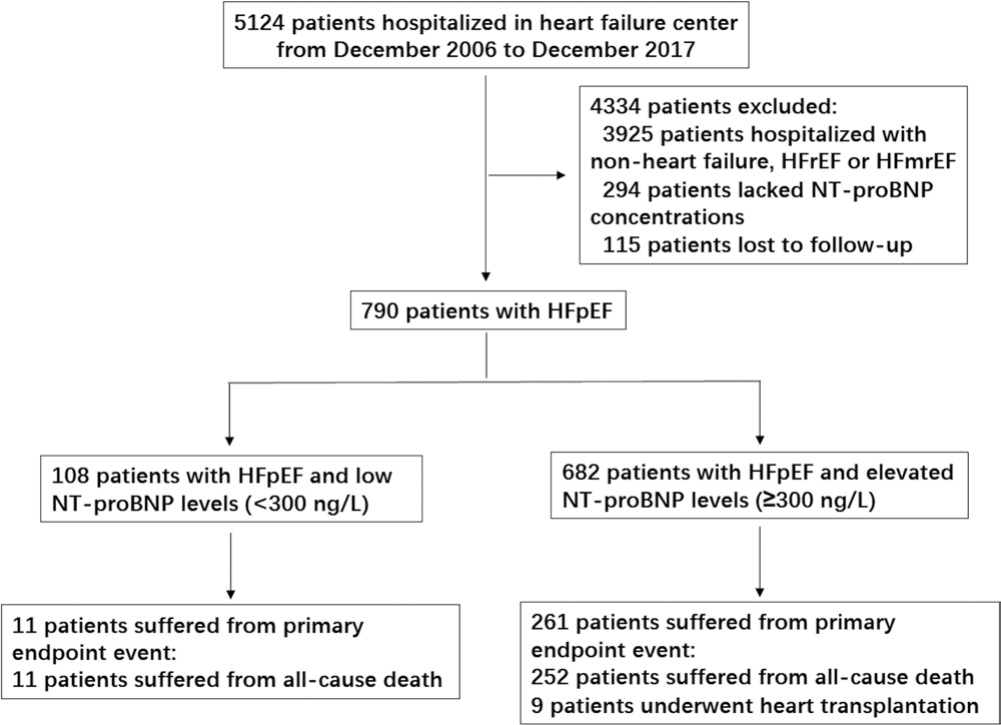
Study flow diagram. This chart showed patients flow regarding enrollment and outcomes assessed for the study. HFpEF = heart failure with preserved ejection fraction, HFmrEF = heart failure with mildly reduced ejection fraction, HFrEF = heart failure with reduced ejection fraction, NT-proBNP = N-terminal pro-B-type natriuretic peptide.

From December 2006 to December 2017, 790 consecutive patients hospitalized with HFpEF in the heart failure center of Fuwai Hospital were enrolled, including 108 patients (13.7%) with low NT-proBNP levels and 682 patients with elevated NT-proBNP levels (Fig. 1).

Compared to those with elevated NT-proBNP levels, patients with HFpEF and low NT-proBNP levels were younger and had less atrial fibrillation and NYHA functional class III–IV, higher BMI and diastolic blood pressure, lower heart rate, and shorter hospitalization duration. On echocardiographic characteristics, patients with HFpEF and low NT-proBNP levels had smaller left atrial diameter compared to those with elevated NT-proBNP levels. On laboratory characteristics, patients with HFpEF and low NT-proBNP levels had higher levels of hemoglobin, albumin, sodium, triglyceride, and eGFR, and lower levels of blood urea nitrogen, creatinine, and high-sensitivity C-reactive protein compared to those with elevated NT-proBNP levels. On prescribed medication, patients with HFpEF and low NT-proBNP levels had a higher rate of angiotensin converting enzyme inhibitor/angiotensin receptor blocker, and a lower rate of mineralocorticoid receptor antagonist than those with elevated NT-proBNP levels (Table 1).

**Table 1:** Baseline clinical characteristics of the study population.

	NT-proBNP < 300 (n = 108)	NT-proBNP ≥ 300 (n = 682)	*P*-value
Age (years)	55.5 (44.3,67.0)	65.0 (55.0,74.0)	<.001
Female n (%)	36 (33.3%)	290 (42.5%)	.072
BMI (kg/m^2^)	26.4 (23.2,28.7)	24.2 (21.5,26.7)	<.001
Heart rate (bpm)	70.5 (64.0,83.0)	76.0 (64.0,88.0)	.046
SBP (mm Hg)	126.0 (116.3137.8)	124.0 (110.0,140.0)	.113
DBP (mm Hg)	73.0 (67.0,80.8)	70.0 (60.0,79.0)	.002
NYHA functional class III–IV n (%)	34 (31.8%)	451 (69.2%)	<.001
Hospitalization duration (days)	8.5 (6.0,13.0)	11.0 (8.0,17.0)	<.001
*Comorbidities*			
Hypertension n (%)	63 (58.3%)	373 (54.7%)	.480
Diabetes mellitus n (%)	33 (30.6%)	186 (27.3%)	.479
Coronary artery disease n (%)	42 (38.9%)	260 (38.1%)	.879
Atrial fibrillation n (%)	26 (24.1%)	333 (48.8%)	<.001
*Medication*			
ACEI/ARB n (%)	68 (63.0%)	290 (42.5%)	<.001
Beta blocker n (%)	89 (82.4%)	512 (75.1%)	.097
MRA n (%)	37 (34.3%)	307 (45.0%)	.036
Diuretic* n (%)	79 (73.1%)	518 (76.0%)	.528
*Echocardiographic measurements*			
LAD (mm)	38.0 (34.0,43.0)	43.0 (38.0,50.3)	<.001
LVEDD (mm)	50.0 (46.0,54.8)	49.0 (45.0,56.0)	.423
LVEF (%)	60.0 (55.0,63.9)	60.0 (55.0,62.0)	.376
*Laboratory measurements*			
White blood cell (*10^9^/L)	7.1 (5.4,8.3)	6.7 (5.3,8.4)	.796
Hemoglobin (g/L)	140.5 (126.0,154.0)	126.0 (112.0,143.0)	<.001
Albumin (g/L)	42.9 (38.6,45.7)	38.9 (35.1,42.4)	<.001
Alanine aminotransferase (U/L)	21.0 (14.0,29.0)	19.0 (12.0,29.0)	.193
Potassium (mmol/L)	3.9 (3.7,4.1)	3.9 (3.7,4.3)	.312
Sodium (mmol/L)	138.1 (136.2140.8)	137.6 (135.0,140.0)	.045
Triglyceride (mmol/L)	1.6 (1.1,2.3)	1.2 (0.9,1.7)	<.001
Total cholesterol (mmol/L)	4.0 (3.3,5.0)	3.9 (3.2,4.6)	.176
LDL-C (mmol/L)	2.2 (1.8,3.1)	2.3 (1.8,2.8)	.314
Creatinine (umol/L)	81.2 (68.8,98.4)	90.8 (74.9113.8)	<.001
eGFR (mL/min/1.73 m^2^, CKD-EPI)	82.2 (64.3,95.7)	65.9 (49.1,82.4)	<.001
Blood urea nitrogen (mmol/L)	5.9 (4.6,8.0)	7.5 (5.7,10.1)	<.001
Uric acid (umol/L)	398.5 (315.3482.9)	401.7 (316.9524.0)	.276
hs-CRP (mg/L)	1.7 (0.9,4.5)	3.6 (1.6,10.7)	<.001
NT-proBNP (ng/L)	130.0 (60.3222.5)	1490.0 (792.9,3322.3)	<.001

ACEI = angiotensin converting enzyme inhibitor, ARB = angiotensin receptor blocker, BMI = body mass index, CKD-EPI = chronic kidney disease epidemiology collaboration, DBP = diastolic blood pressure, eGFR = estimated glomerular filtration rate, hs-CRP = high sensitivity C reactive protein, LAD = left atrial diameter, LDL-C = low density lipoprotein cholesterol, LVEDD = left ventricular end diastolic diameter, LVEF = left ventricular ejection fraction, MRA = mineralocorticoid receptor antagonist, NT-proBNP = N-terminal pro-B type natriuretic peptide, NYHA = New York Heart Association, SBP = systolic blood pressure.

* MRA was excluded.

### 3.1. Independent predictors of low NT-proBNP levels in patients with HFpEF

Covariates were enrolled including age, sex, BMI, atrial fibrillation, NYHA functional class, hemoglobin, albumin, sodium, eGFR, and high-sensitivity C-reactive protein. Multivariate logistic regression analysis showed that BMI and albumin were independent predictors for low NT-proBNP levels in patients with HFpEF. However, age, atrial fibrillation, and NYHA functional class were independent predictors against low NT-proBNP levels in patients with HFpEF (Table 2).

**Table 2:** Multivariate logistic regression analysis for independent predictors of low NT-proBNP levels in patients with HFpEF.

	Univariate model		Multivariate model	
	OR (95%CI)	*P*-value	OR (95%CI)	*P*-value
Age	0.97 (0.96,0.98)	<.001	0.98 (0.96,1.00)	.018
Female	0.68 (0.44,1.04)	.073	1.58 (0.90,2.78)	.109
BMI	1.10 (1.05,1.16)	<.001	1.10 (1.04,1.17)	.001
Atrial fibrillation	0.33 (0.21,0.53)	<.001	0.42 (0.24,0.73)	.002
NYHA functional class III–IV	0.21 (0.13,0.32)	<.001	0.33 (0.20,0.57)	<.001
Hemoglobin	1.02 (1.01,1.03)	<.001	1.01 (0.99,1.02)	.488
Albumin	1.13 (1.08,1.18)	<.001	1.09 (1.03,1.16)	.005
Sodium	1.05 (1.00,1.11)	.052	0.97 (0.91,1.04)	.428
eGFR < 60 mL/min/1.73 m^2^	0.37 (0.23,0.61)	<.001	0.67 (0.36,1.23)	.193
hs-CRP	0.89 (0.85,0.94)	<.001	0.97 (0.91,1.03)	.289

BMI = body mass index, CI = confidence interval, eGFR = estimated glomerular filtration rate, HFpEF = heart failure with preserved ejection fraction, hs-CRP = high sensitivity C reactive protein, NT-proBNP = N-terminal pro-B type natriuretic peptide, NYHA = New York Heart Association, OR = odds ratio.

### 3.2. Clinical outcomes of patients with HFpEF and low NT-proBNP levels

During the median follow-up duration of 1103 days (interquartile range: 426–2045 days), 11 patients (10.2%) suffered from primary endpoint event in patients with HFpEF and low NT-proBNP levels, and all were all-cause death. In patients with HFpEF and elevated NT-proBNP levels, 261 patients (38.3%) suffered from primary endpoint event, including 252 all-cause deaths (96.6%) and 9 heart transplantations (3.4%). 15 patients suffered from all-cause death during hospitalization, and all came from those with elevated NT-proBNP levels. No patient underwent heart transplantation during hospitalization (Fig. 1).

**Figure 2.**
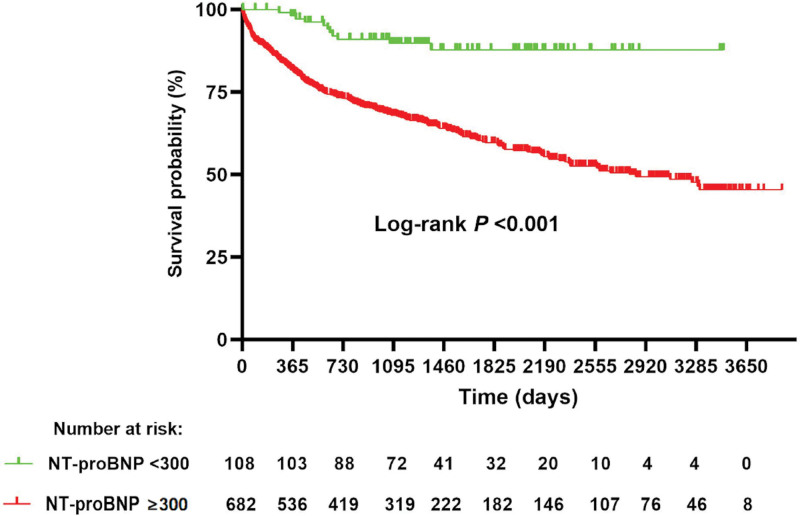
Kaplan–Meier curves of all-cause death or heart transplantation between low NT-proBNP group (<300 ng/L) and elevated NT-proBNP group (≥300 ng/L). NT-proBNP = N-terminal pro-B-type natriuretic peptide.

Adjusted for age, sex, BMI, and NYHA functional class, multivariate Cox regression analysis showed that patients with HFpEF and elevated NT-proBNP levels had a higher risk of all-cause death or heart transplantation compared to those with low NT-proBNP levels (adjusted HR [95%CI]: 2.36 [1.24,4.49], *P* = .009) (Table 3). Additionally, Kaplan–Meier analysis further demonstrated that patients with HFpEF and elevated NT-proBNP levels had a higher rate of all-cause death or heart transplantation than those with low NT-proBNP levels (*P* < .001) (Fig. 2).

**Table 3:** Multivariate Cox regression analysis for primary endpoint event in patients with HFpEF.

	Univariate model		Multivariate model	
	HR(95%CI)	*P*-value	HR (95%CI)	*P*-value
Age	1.03 (1.02,1.04)	<.001	1.02 (1.01,1.03)	<.001
Female	1.17 (0.92,1.49)	.191	0.87 (0.67,1.13)	.287
BMI	0.92 (0.89,0.95)	<.001	0.94 (0.91,0.98)	.001
NYHA functional class III-IV	4.62 (3.23,6.59)	<.001	3.64 (2.51,5.27)	<.001
Elevated NT-proBNP vs. low NT-proBNP	4.35 (2.38,7.95)	<.001	2.36 (1.24,4.49)	.009

BMI = body mass index, CI = confidence interval, HFpEF = heart failure with preserved ejection fraction, HR = hazard ratio, NT-proBNP = N-terminal pro-B type natriuretic peptide, NYHA = New York Heart Association.

### 3.3. Subgroup analyses

**Figure 3.**
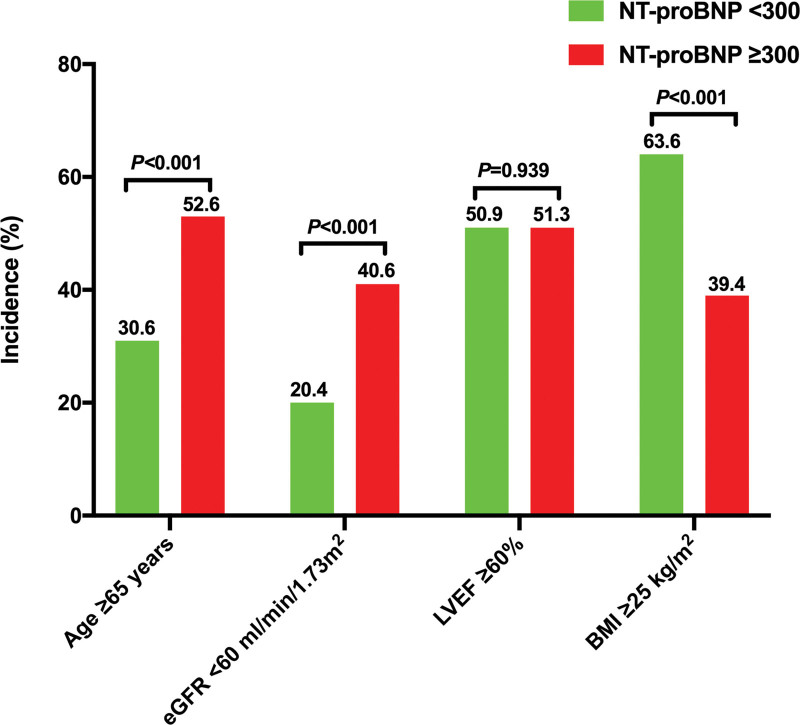
Rates of patients with age ≥ 65 years, patients with eGFR < 60 mL/min/1.73 m^2^, patients with LVEF ≥ 60%, and patients with BMI ≥ 25 kg/m^2^ between low NT-proBNP group (<300 ng/L) and elevated NT-proBNP group (≥300 ng/L). NT-proBNP = N-terminal pro-B-type natriuretic peptide, eGFR = estimated glomerular filtration rate, LVEF = left ventricular ejection fraction, BMI = body mass index.

Compared to those with elevated NT-proBNP levels, patients with HFpEF and low NT-proBNP levels had lower rates of age ≥ 65 years and eGFR < 60 mL/min/1.73 m^2^. Overweight or obesity (BMI ≥ 25 kg/m^2^) was more common in patients with HFpEF and low NT-proBNP levels than those with elevated NT-proBNP levels. However, the rate of LVEF ≥ 60% was comparable between the 2 groups (Fig. 3).

**Figure 4.**
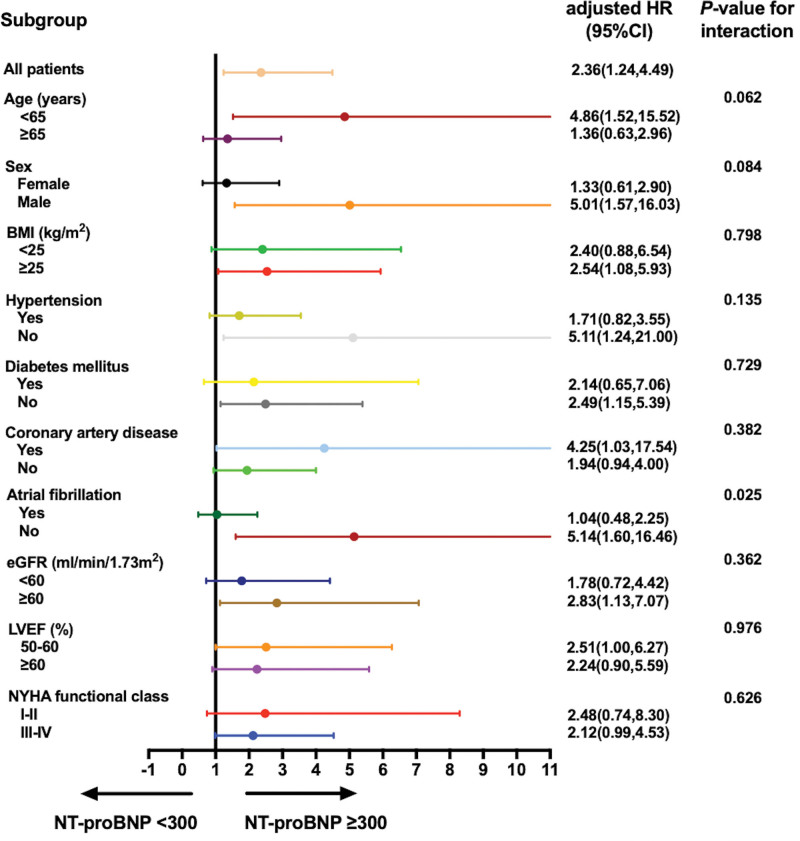
Subgroup analyses of the associations between baseline NT-proBNP (elevated NT-proBNP group vs low NT-proBNP group) and risk of all-cause death or heart transplantation. Adjusted for age, sex, BMI, and NYHA functional class. BMI = body mass index, CI = confidence interval, eGFR = estimated glomerular filtration rate, HR = hazard ratio, LVEF = left ventricular ejection fraction, NT-proBNP = N-terminal pro-B-type natriuretic peptide, NYHA = New York Heart Association.

Stratified analyses were conducted by age (<65, and ≥65 years), sex, BMI (<25, and ≥25 kg/m^2^), hypertension, diabetes mellitus, coronary artery disease, atrial fibrillation, eGFR (<60, and ≥60 mL/min/1.73 m^2^), LVEF (50–60%, and ≥60%), and NYHA functional class (I–II and III–IV). Adjusted covariates included age, sex, BMI, and NYHA functional class, and stratified analyses showed that the association between NT-proBNP (elevated NT-proBNP group vs low NT-proBNP group) and risk of all-cause death or heart transplantation was stronger in non-atrial fibrillation patients (adjusted HR [95%CI]: 5.14 [1.60,16.46]) than in atrial fibrillation patients (adjusted HR [95%CI]: 1.04 [0.48,2.25], *P* value for interaction = 0.025). Furthermore, the association between NT-proBNP (elevated NT-proBNP group vs. low NT-proBNP group) and risk of all-cause death or heart transplantation was stronger in younger patients (adjusted HR [95%CI]: 4.86 [1.52,15.52]) than in older patients (adjusted HR [95%CI]: 1.36 [0.63,2.96], *P* value for interaction = .062), as well as stronger in male patients (adjusted HR [95%CI]: 5.01 [1.57,16.03]) than in female patients (adjusted HR [95%CI]: 1.33 [0.61,2.90], *P* value for interaction = .084). However, both subgroups only reached borderline significant (Fig. 4).

## 4. Discussion

The main findings of this study are as follows: (1) the incidence of low NT-proBNP levels in patients hospitalized with HFpEF was 13.7%; (2) age, BMI, atrial fibrillation, NYHA functional class, and albumin were independent predictors of low NT-proBNP levels in patients with HFpEF; (3) patients with HFpEF and low NT-proBNP levels had a lower risk of all-cause death or heart transplantation compared to those with elevated NT-proBNP levels, particularly in younger, male, and non-atrial fibrillation patients.

Studies on the rate of low natriuretic peptide levels in patients with HFpEF are scarce, and the accurate incidence of low natriuretic peptide levels in patients with HFpEF is still uncertain. A retrospective study reported that the rate of low B-type natriuretic peptide (BNP) levels (<100 ng/L) in patients hospitalized with HFpEF was 13%.^[12]^ COACH study reported that the rate of low BNP levels in patients hospitalized with HFpEF was 19%.^[13]^ The incidence of low NT-proBNP levels in patients hospitalized with HFpEF was 13.7% in our study, which was consistent with previous studies. A substantial proportion of symptomatic patients with HFpEF present with low natriuretic peptide levels. Therefore, low natriuretic peptide levels could not rule out heart failure in some symptomatic patients with HFpEF. More attention should be paid to patients with HFpEF and low natriuretic peptide levels, and synthetic evaluation should be taken to assess whether these patients present with heart failure or not.

Studies reported that BMI, chronic kidney disease, and atrial fibrillation were independent predictors of low BNP levels in patients with HFpEF.^[14,15]^ Multivariate logistic regression analysis showed that BMI and atrial fibrillation were independent predictors of low NT-proBNP levels in patients with HFpEF in our study, which were consistent with previous studies. Univariate logistic regression analysis showed that eGFR was associated with low NT-proBNP levels in patients with HFpEF. However, multivariate logistic regression analysis showed that eGFR was not an independent predictor of low NT-proBNP levels in patients with HFpEF in our study, which may be associated with small sample in low NT-proBNP group. Furthermore, we found that age, NYHA functional class, and albumin were independent predictors of low NT-proBNP levels in patients with HFpEF. Natriuretic peptide increases with age, associated with decreased metabolism and increased production.^[16]^ NYHA functional class and albumin are marks of severity in patients with HFpEF,^[17]^ which in turn affect the NT-proBNP levels. Further study is needed to clarify the associated predictors of low natriuretic peptide levels in patients with HFpEF.

Studies reported that patients with HFpEF and elevated natriuretic peptide levels had a higher rate of mortality or heart failure rehospitalization compared to those with low natriuretic peptide levels.^[10,14,18,19]^ Our study also showed that patients with HFpEF and elevated NT-proBNP levels had a higher risk of all-cause death or heart transplantation compared to those with low NT-proBNP levels, which was consistent with previous studies. However, COACH study reported that the primary and secondary outcomes were comparable between the 2 groups.^[13]^ Sakane K, et al also reported that the prognosis of patients with HFpEF and low BNP levels was as poor as those with elevated BNP levels.^[15]^ Further study is needed to clarify the association between low natriuretic peptide levels and clinical outcomes in patients with HFpEF.

Another interesting finding is that the association between NT-proBNP (elevated NT-proBNP group vs. low NT-proBNP group) and risk of all-cause death or heart transplantation was stronger in younger and non-atrial fibrillation patients than in older and atrial fibrillation patients. However, the subgroup of age only reached borderline significant, which may be associated with small sample in low NT-proBNP group. One study reported that BNP (as continuous variable) was an independent predictor of cardiac events in younger patients with heart failure, but not in older patients with heart failure.^[20]^ However, our study showed that the prognostic value of NT-proBNP (as continuous variable) in HFpEF was comparable across subgroups of age and atrial fibrillation (Table S1, Supplemental Digital Content, http://links.lww.com/MD/K891), which was consistent with previous study.^[21]^ Atrial fibrillation and advanced age are important causes of elevated natriuretic peptide levels,^[16]^ and associated with adverse events in HFpEF.^[22,23]^ Therefore, atrial fibrillation and older patients may need higher natriuretic peptide levels for prognosis evaluation of HFpEF than non-atrial fibrillation and younger patients. Our study also showed that low NT-proBNP levels had a lower risk of all-cause death or heart transplantation in non-atrial fibrillation and younger patients, and the cutoff values of NT-proBNP in atrial fibrillation and older patients were higher than those in non-atrial fibrillation and younger patients (Fig. S1A and B, Supplemental Digital Content, http://links.lww.com/MD/K890), which may be associated with the lower risk of adverse events in non-atrial fibrillation and younger patients. Furthermore, our study found that the association between NT-proBNP and risk of all-cause death or heart transplantation was stronger in male patients than in female patients. However, it only reached borderline significant, which may be associated with small sample in low NT-proBNP group. Nakada Y, et al also reported that high BNP group had worse prognosis than low BNP group in male patients but not in female patients.^[24]^ And we found that low NT-proBNP levels had a lower risk of all-cause death or heart transplantation in male patients, and NT-proBNP was a more valuable biomarker for prognosis evaluation of HFpEF in male patients than in female patients (Table S1, Supplemental Digital Content, http://links.lww.com/MD/K891 and Fig. S1C, Supplemental Digital Content, http://links.lww.com/MD/K890), which was consistent with previous studies.^[25,26]^ Further study is needed to clarify the clinical outcomes between patients with HFpEF and low natriuretic peptide levels and those with elevated natriuretic peptide levels in different subgroups.

There are several limitations to our study. First, some clinical characteristics like left atrial volume index, E/A ratio, and E/e′ ratio were lacking, as they were not recorded for majority of patients with heart failure in echocardiographic reports. However, all enrolled patients with HFpEF had objective evidence of left ventricular diastolic dysfunction or increased left ventricular filling pressure (e.g., E/e′ ratio ≥ 13, E/A ratio < 1, estimated pulmonary artery systolic pressure > 35 mm Hg, tricuspid regurgitation velocity > 2.8 m/s, elevated NT-proBNP level, or pulmonary congestion). Besides, patients who lacked the NT-proBNP levels or lost to follow-up were excluded in this study, which leads to a selection bias. Finally, this is a retrospective and single-center study, the sample is relatively small, and the primary endpoint event in low NT-proBNP group is relatively low, which may decrease the confidence of the conclusion. These findings remain to be tested with prospective, multicenter and large sample trials.

## 5. Conclusion

Low NT-proBNP levels were common in patients hospitalized with HFpEF. Patients with HFpEF and low NT-proBNP levels had a better prognosis than those with elevated NT-proBNP levels, particularly in younger, male, and non-atrial fibrillation patients.

## Author contributions

**Conceptualization:** Yu-Yi Chen, Jian Zhang, Yu-Hui Zhang.

**Data curation:** Yu-Yi Chen, Lin Liang, Peng-Chao Tian, Jia-Yu Feng, Li-Yan Huang, Bo-Ping Huang, Xue-Mei Zhao, Yi-Hang Wu, Jing Wang, Jing-Yuan Guan, Xin-Qing Li.

**Formal analysis:** Yu-Yi Chen, Jia-Yu Feng.

**Funding acquisition:** Jian Zhang.

**Investigation:** Yu-Yi Chen, Jian Zhang, Yu-Hui Zhang.

**Methodology:** Yu-Yi Chen, Jia-Yu Feng.

**Project administration:** Jian Zhang, Yu-Hui Zhang.

**Supervision:** Jian Zhang, Yu-Hui Zhang.

**Visualization:** Yu-Yi Chen.

**Writing – original draft:** Yu-Yi Chen.

**Writing – review & editing:** Yu-Yi Chen, Jian Zhang, Yu-Hui Zhang.

## Supplementary Material
