# Supplementary material for: Clinical characteristics and outcomes of patients hospitalized with heart failure with preserved ejection fraction and low NT-proBNP levels
Source: Medicine (Baltimore). 2023 Nov 24;102(47):e36351. doi: 10.1097/MD.0000000000036351 (PMC10681576; doi:10.1097/MD.0000000000036351)
Supplement: Supplementary file 1 [file medi-102-e36351-s001.docx]

**Supplementary Table 1. Subgroup analyses of the associations between baseline NT-proBNP and risk of all-cause death or heart transplantation**

|  | Adjusted HR(95%CI) | *P*-value for interaction |
| --- | --- | --- |
| Age |  | 0.393 |
| <65 years | 1.34(1.19,1.51) |  |
| ≥65 years | 1.32(1.19,1.47) |  |
| Sex |  | 0.014 |
| Female | 1.17(1.04,1.32) |  |
| Male | 1.44(1.30,1.60) |  |
| Atrial fibrillation |  | 0.968 |
| Yes | 1.31(1.17,1.47) |  |
| No | 1.31(1.17,1.47) |  |

NT-proBNP was log_2_-transformed. Adjusted for age, sex, BMI and NYHA functional class.

NT-proBNP = N-terminal pro-B-type natriuretic peptide, BMI = body mass index, NYHA = New York Heart Association, HR = hazard ratio, CI = confidence interval.
