# Supplementary figures and images for: Clinical characteristics and outcomes of patients hospitalized with heart failure with preserved ejection fraction and low NT-proBNP levels
Source: Medicine (Baltimore). 2023 Nov 24;102(47):e36351. doi: 10.1097/MD.0000000000036351 (PMC10681576; doi:10.1097/MD.0000000000036351)

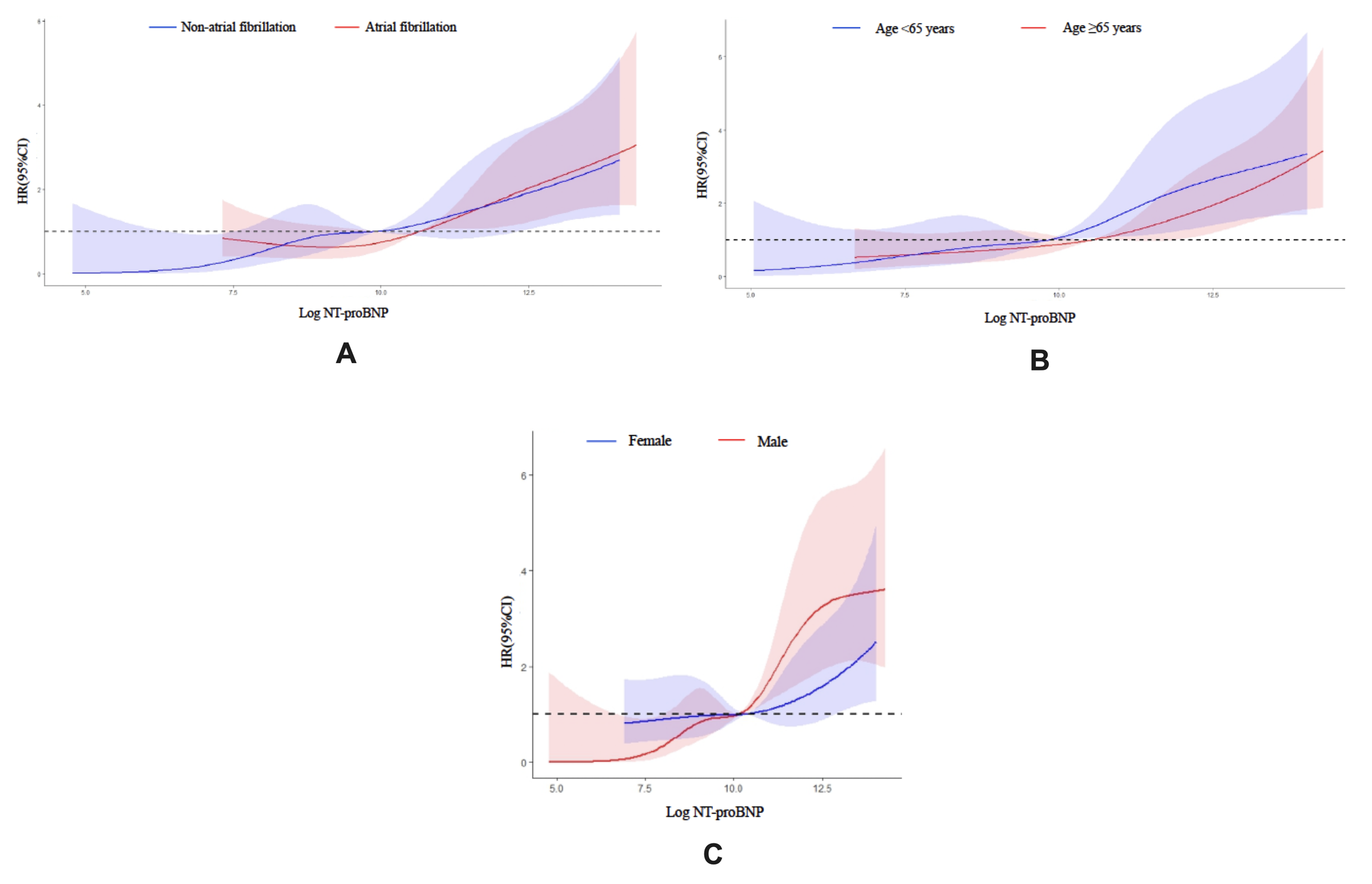

Supplement: Supplementary file 2 [file medi-102-e36351-s002.tiff]
